# Supplementary figures and images for: Influence of nutrient status on the response of the diatom Phaeodactylum tricornutum to oil and dispersant
Source: PLoS One. 2021 Dec 1;16(12):e0259506. doi: 10.1371/journal.pone.0259506 (PMC8635359; doi:10.1371/journal.pone.0259506)

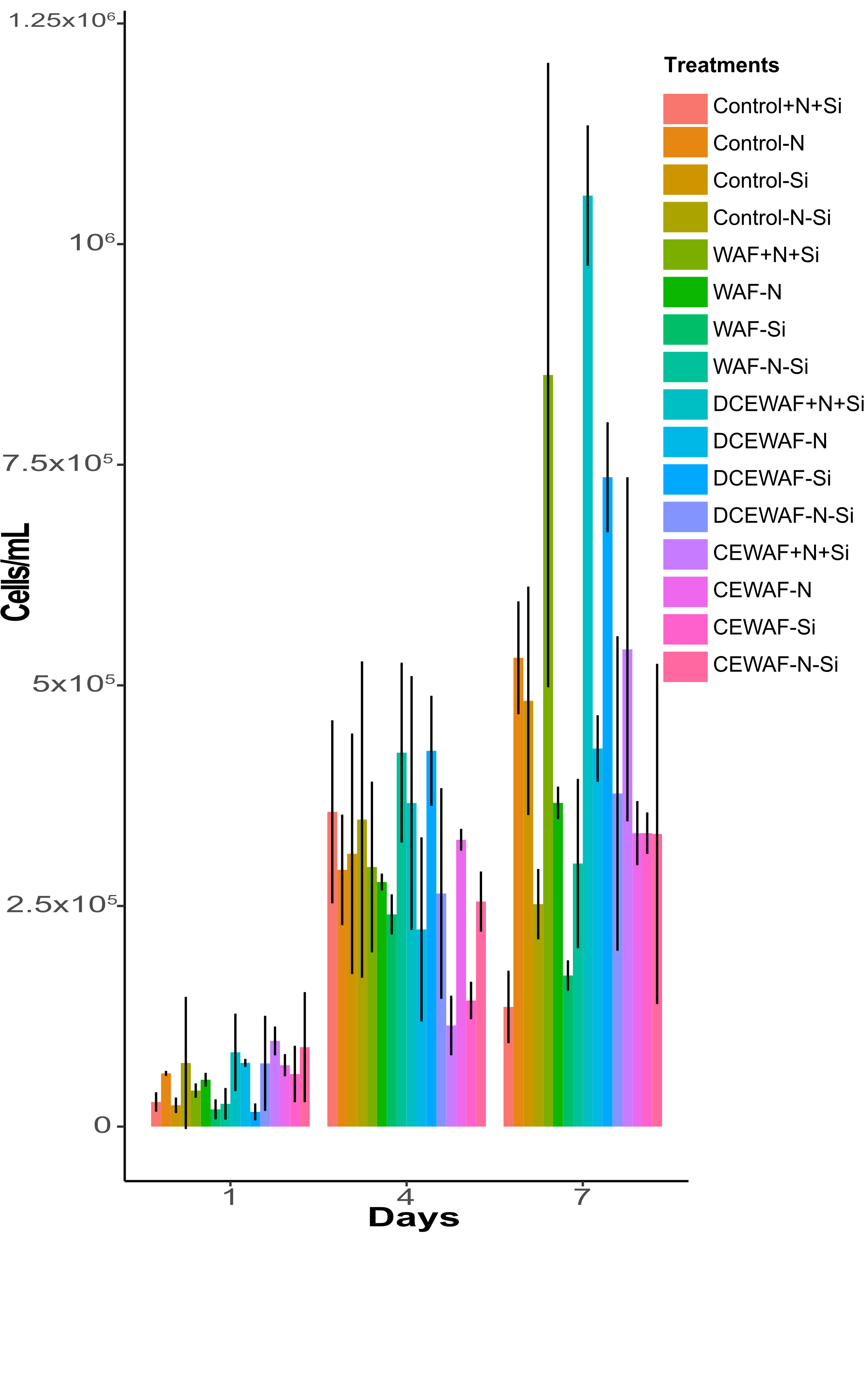

Supplement: S1 Fig — The symbols–N, -Si, -N-Si, and +N+Si indicate nitrogen limited, silica limited, both nitrogen and silica limited and nitrogen and silica replete treatments. (TIFF) [file pone.0259506.s001.tiff]

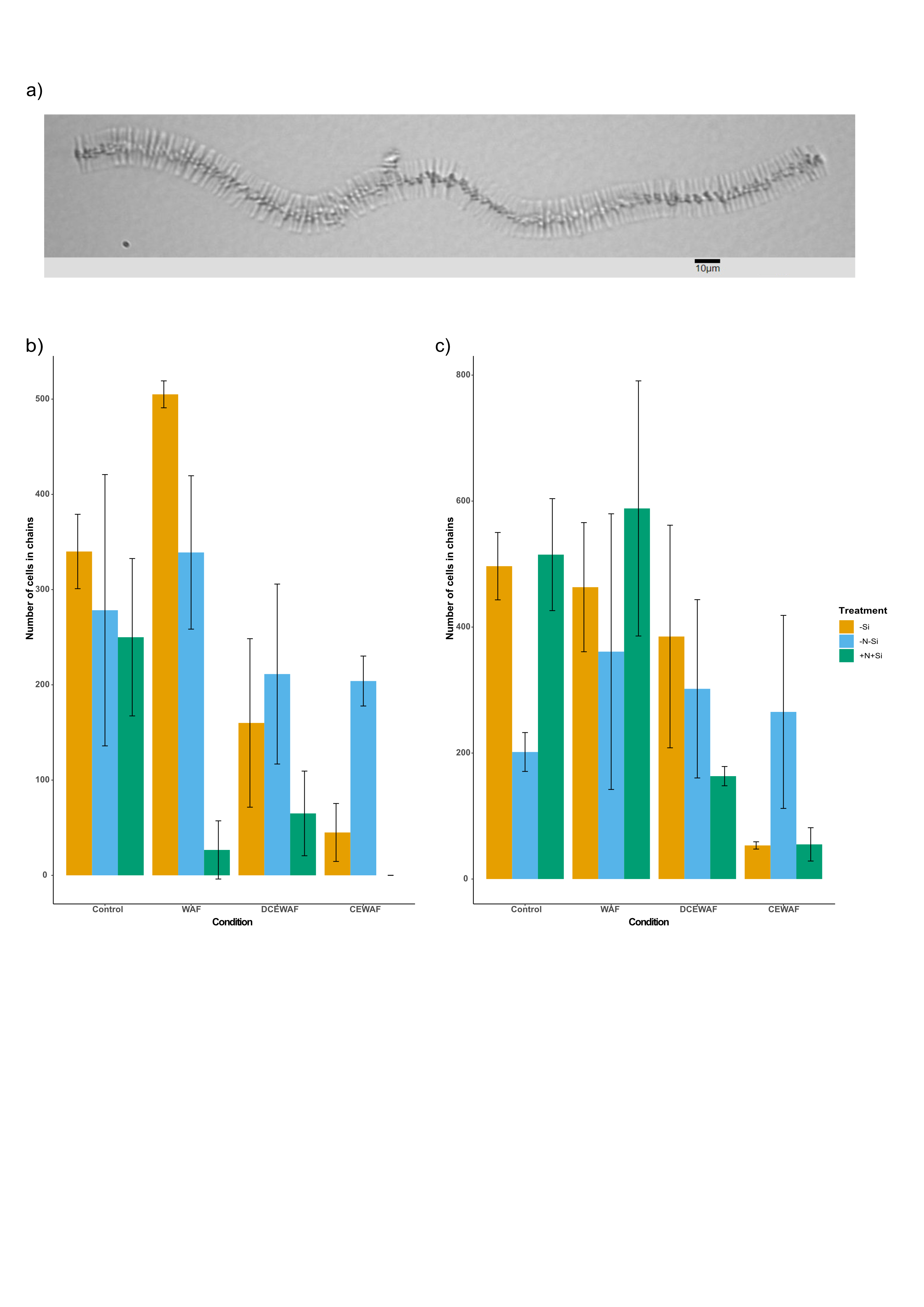

Supplement: S2 Fig — a) average number of cells in chains on Day 1, b) average number of cells in chains on Day 4, c) average number of cells in chains on Day 7 (± standard deviation) under different treatments and conditions (n = 3). The symbols–N, -Si, -N-Si, and +N+Si indicate nitrogen limited, silica limited, both nitrogen and silica limited and nitrogen and silica replete treatments. (TIFF) [file pone.0259506.s002.tiff]

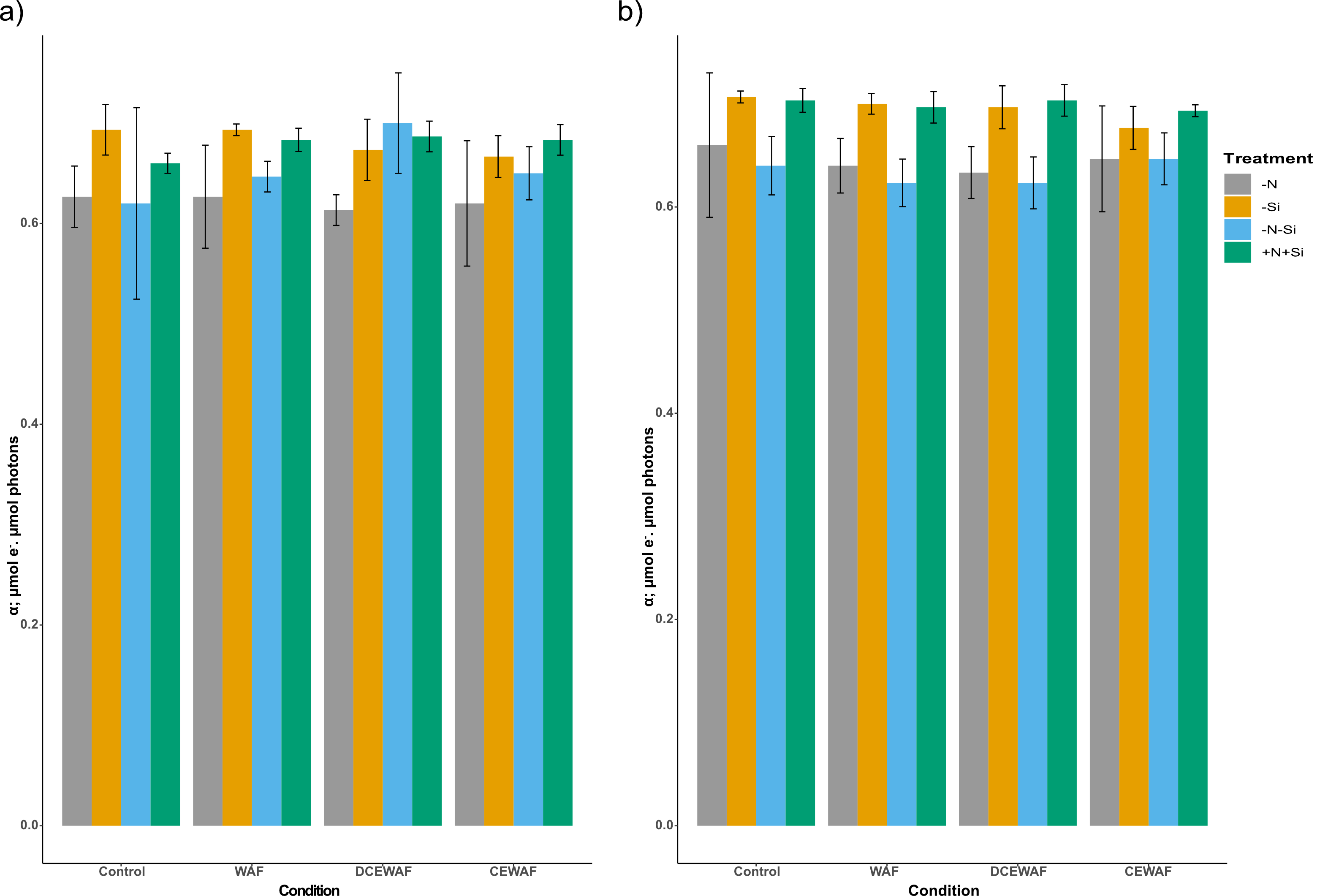

Supplement: S3 Fig — a) average light harvesting ability on Day 1 (α; μmol e-. μmol photons), b) average light harvesting ability on Day 4 (α; μmol e-. μmol photons) (± standard deviation) under different treatments and conditions (n = 3). The symbols–N, -Si, -N-Si, and +N+Si indicate nitrogen limited, silica limited, both nitrogen and silica limited and nitrogen and silica replete treatments. (TIFF) [file pone.0259506.s003.tiff]

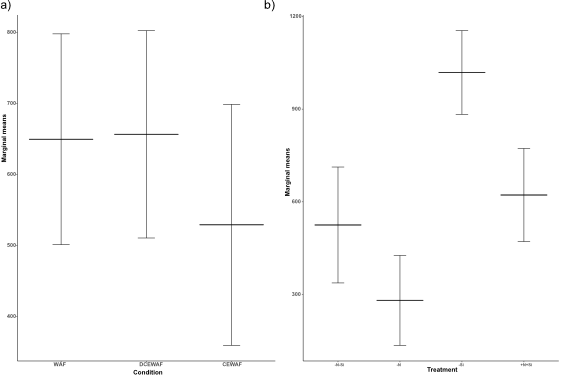

Supplement: S4 Fig — a) Marginal effects of different conditions on relative growth of P. tricornutum, b) Marginal effects of different nutrient treatments on relative growth of P. tricornutum. The symbols–N, -Si, -N-Si, and +N+Si indicate nitrogen limited, silica limited, both nitrogen and silica limited and nitrogen and silica replete treatments. (TIFF) [file pone.0259506.s004.tiff]
