# Supplementary material for: Influence of nutrient status on the response of the diatom Phaeodactylum tricornutum to oil and dispersant
Source: PLoS One. 2021 Dec 1;16(12):e0259506. doi: 10.1371/journal.pone.0259506 (PMC8635359; doi:10.1371/journal.pone.0259506)
Supplement: S1 Table — (DOCX) [file pone.0259506.s005.docx]

**List of Supplementary Tables:**

Supplementary Table 1: Summary of generalized linear modelling of relative growth vs oil concentration with interaction of different treatments and conditions as factorial variables.

|  | **Coefficients:**  **Estimate** | **Std.Error** | **t value** | **Pr(>\|t\|)** |  |
| --- | --- | --- | --- | --- | --- |
| **(Intercept)** | 1162.593 | 182.205 | 6.381 | 1.51e-09 | *** |
| **Oil concentration** | -30.856 | 7.221 | -4.273 | 3.16e-05 | *** |
| **DCEWAF** | -722.371 | 258.918 | -2.790 | 0.00585 | ** |
| **CEWAF** | -482.989 | 276.527 | -1.747 | 0.08245 | . |
| **-N** | -639.287 | 225.386 | -2.836 | 0.00510 | ** |
| **-Si** | -145.071 | 218.011 | -0.665 | 0.50665 |  |
| **+N+Si** | -325.096 | 223.853 | -1.452 | 0.14821 |  |
| **DCEWAF : -N** | 745.040 | 327.421 | 2.275 | 0.02408 | * |
| **CEWAF : -N** | 440.665 | 329.667 | 1.337 | 0.18305 |  |
| **DCEWAF : -Si** | 1560.160 | 308.134 | 5.063 | 1.03e-06 | *** |
| **CEWAF : -Si** | 355.697 | 311.818 | 1.141 | 0.25554 |  |
| **DCEWAF : +N+Si** | 612.158 | 313.540 | 1.952 | 0.05248 | . |
| **CEWAF : +N+Si** | 654.161 | 329.894 | 1.983 | 0.04893 | * |
